# Bee venom ameliorates lipopolysaccharide-induced memory loss by preventing NF-kappaB pathway

**DOI:** 10.1186/s12974-015-0344-2

**Published:** 2015-06-26

**Authors:** Sun Mi Gu, Mi Hee Park, Chul Ju Hwang, Ho Sueb Song, Ung Soo Lee, Sang Bae Han, Ki Wan Oh, Young Wan Ham, Min Jong Song, Dong Ju Son, Jin Tae Hong

**Affiliations:** College of Pharmacy and Medical Research Center, Chungbuk National University, 194-31 Osongsaemgmyeong 1-ro, Osong-eup, Heungdeok-gu, Cheongju, Chungbuk 361-951 Republic of Korea; College of Oriental Medicine, Gachon University, San 65, Bokjeong-dong, Sujeong-gu, Seongnam, Gyeonggii-do 461-701 Republic of Korea; Department of Food Science & Technology, Korea National University of Transportation, Jeungpyeong, 368-701 Republic of Korea; Department of Chemistry, Utah Valley University, 800 W University Pkwy, Orem, UT 84058 USA; Department of Obstetrics and Gynecology, Daejeon St. Mary’s Hospital, College of Medicine, The Catholic University of Korea, 64 Daeheung-ro, Jung gu Daejeon, 301-723 Republic of Korea

**Keywords:** Alzheimer’s disease, Beta-amyloid, Bee venom, Nuclear factor kappaB, Lipopolysaccharide, Neuroinflammation

## Abstract

**Background:**

Accumulation of beta-amyloid and neuroinflammation trigger Alzheimer’s disease. We previously found that lipopolysaccharide (LPS) caused neuroinflammation with concomitant accumulation of beta-amyloid peptides leading to memory loss. A variety of anti-inflammatory compounds inhibiting nuclear factor kappaB (NF-κB) activation have showed efficacy to hinder neuroinflammation and amyloidogenesis. We also found that bee venom (BV) inhibits NF-κB.

**Methods:**

A mouse model of LPS-induced memory loss used administration of BV (0.8 and 1.6 μg/kg/day, i.p.) to ICR mice for 7 days before injection of LPS (2.5 mg/kg/day, i.p.). Memory loss was assessed using a Morris water maze test and passive avoidance test. For in vitro study, we treated BV (0.5, 1, and 2 μg/mL) to astrocytes and microglial BV-2 cells with LPS (1 μg/mL).

**Results:**

We found that BV inhibited LPS-induced memory loss determined by behavioral tests as well as cell death. BV also inhibited LPS-induced increases in the level of beta-amyloid (Aβ), β-and γ-secretases activities, NF-κB and its DNA-binding activity and expression of APP, and BACE1 and neuroinflammation proteins (COX-2, iNOS, GFAP and IBA-1) in the brain and cultured cells. In addition, pull-down assay and molecular modeling showed that BV binds to NF-κB.

**Conclusions:**

BV attenuates LPS-induced amyloidogenesis, neuroinflammation, and therefore memory loss via inhibiting NF-κB signaling pathway. Thus, BV could be useful for treatment of Alzheimer’s disease.

## Introduction

Alzheimer’s disease (AD) is the most common neurodegenerative disease due to loss of neurons in the brain. Typical early symptoms of AD patients are losing their ability to learn new information and then loss of both declarative and non-declarative memory [1]. AD is generally known to be correlated with accumulation of beta-amyloid (Aβ) peptides which is produced from amyloid precursor protein (APP) [2, 3]. APP is decomposed into soluble amyloid precursor protein beta (sAPPβ) and carboxy-terminal 99 amino acid (C99) protein by β-site APP cleaving enzyme (β-secretase; BACE1), and then γ-secretase produces Aβ and amyloid intracellular domain (ACID) from C99 [4]. AD patients have showed a high level of Aβ output and aggregative Aβ [5].

Lipopolysaccharide (LPS) can trigger neuroinflammation and influence amyloidogenesis in neuronal cells. LPS activates inflammatory cells such as astrocytes and microglia cells in the brain, thus enhancing neuroinflammation [6–9]. LPS also induces accumulation of Aβ through increased cleavage of APP by increasing BACE1 and γ-secretase activation [9, 10]. Nuclear factor kappaB (NF-κB) can be activated by LPS, and then induces several inflammatory genes such as tumor necrosis factor-a (TNF-α interleukins (IL-1β, IL-6, and IL-18), inducible nitric oxide synthase (iNOS), and cyclooxygenase-2 (COX-2) [11]. NF-κB also induces decomposition of APP by activating BACE1. BACE1 promoter activity and BACE1 transcription are increased by activating NF-κB because the BACE1 promoter has a NF-κB-binding site [12, 13]. Therefore, inhibiting NF-κB pathways could interrupt generation of Aβ as well as neuroinflammation [14]. A variety of anti-inflammatory compounds inhibiting NF-κB activation, such as (-)-epigallocatechin-3-gallate (EGCG), thiacremonone, and N-adamantyl-4-methylthiazol-2-amine (KHG26693), have showed efficacy to hinder neuroinflammation and amyloidogenesis, and thus reduce memory loss [14–17].

Bee venom (BV) has been used as a traditional cure in oriental countries [18]. Various diseases, such as arthritis, rheumatism, and cancer have been treated by BV [19]. BV consists of various active components, for example: peptides (e.g., melittin, apamin, adolapin, and the mast cell degranulating peptide; MCD), enzymes (e.g., phospholipase A_2_; PLA_2_, hyaluronidase, glucosidase), and biogenic amines (e.g., histamines, dopamine, norepinephrine) [19]. Several research groups have proved the anti-inflammatory effect of BV in several disease models such as a mouse model of Parkinson’s disease, Freund’s adjuvant-induced rheumatoid arthritis (RA) model, and cholecystokinin octapeptide-induced acute pancreatitis rat model [20–22]. BV and its major component melittin inhibited LPS and TNF-α-induced NF-κB activation by interrupting p50 translocation through interaction with sulfhydryl residue of p50 or IκB kinases (IKKα and IKKβ) [23, 24]. Our study also showed that BV prevented LPS-induced nitric oxide (NO) and prostaglandin E_2_ (PGE_2_) production through inactivation of NF-κB in RAW 264.7 cells [25]. In this study, we investigated whether BV inhibits neuroinflammation response and amyloidogenesis through inactivation of NF-κB in vitro and in vivo, and thus reduces memory lose by LPS.

## Materials and methods

### Materials

BV was purchased from You-Miel Bee Venom Ltd. (Hwasoon, Jeonnam, Korea). The composition of the BV was as follows: 45–50 % melittin, 2.5–3 % mast cell degranulating peptide, 12 % phospholipase A2, 1 % lysophospholipase A, 1–1.5 % histidine, 4–5 % 6-pentyl a-pyrone lipids, 0.5 % secarpin, 0.1 % tertiapin, 0.1 % procamine, 1.5–2 % hyaluronidase, 2–3 % amine, 4–5 % carbohydrate, and 19–27 % of others, including protease inhibitor, glucosidase, invertase, acid phosphomonoesterase, dopamine, norepinephrine, and unknown amino acids, with 99.5 % purity. Melittin was purchased from Sigma-Aldrich (St. Louis, MO).

### Animals and treatments

**Fig. 1:**
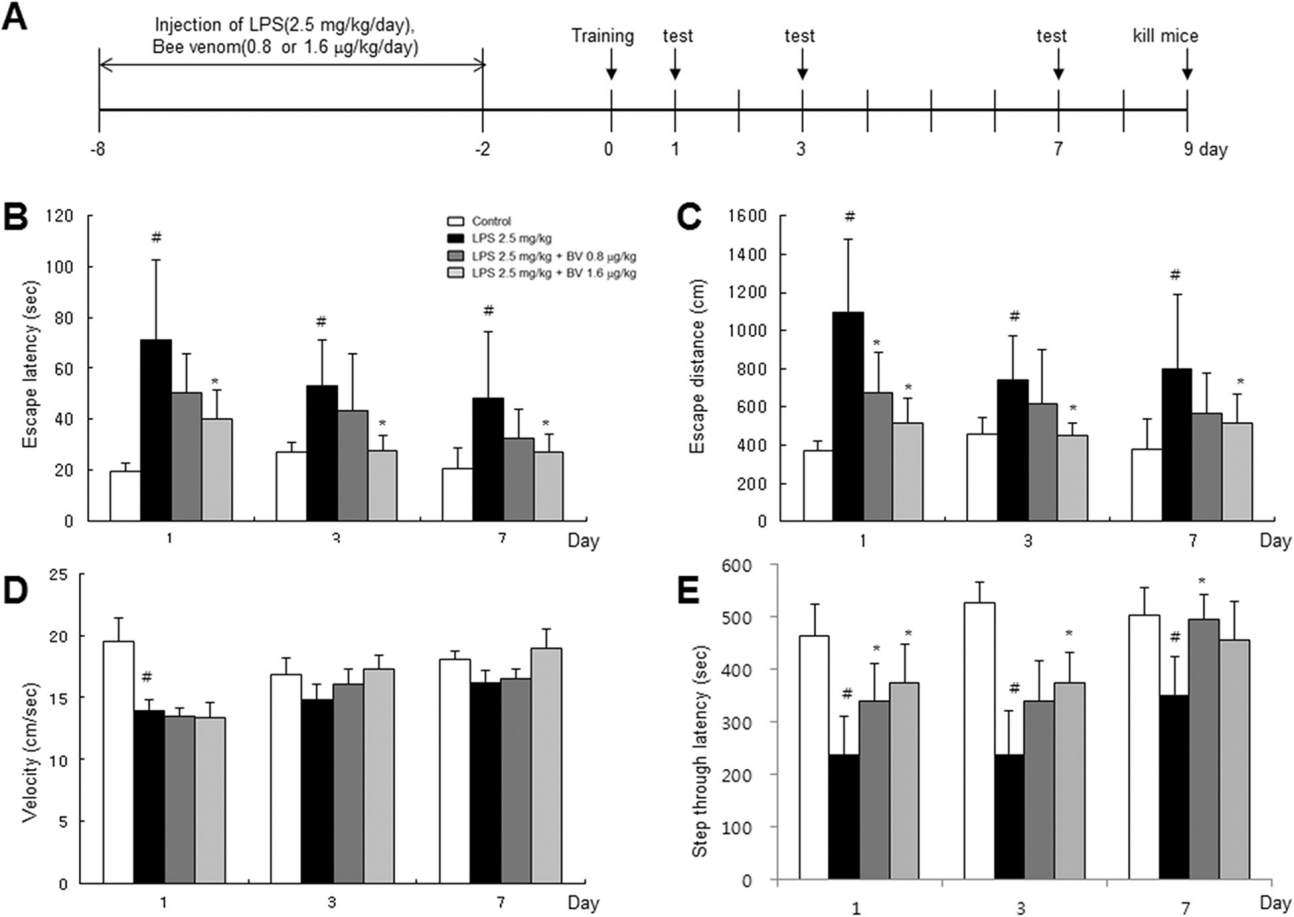
Timeline depicting the treatment of BV and assessments of cognitive functions of mice (**a**). *Arrow heads* represent days on which acquisition tests were conducted. Inhibitory effect of BV on LPS-induced memory defects. Mice were treated with BV (0.8 and 1.6 μg/kg, i.p.) after 20-min treatment of LPS (2.5 mg/kg, i.p.). The Morris water maze tests and passive avoidance tests were performed as described in the Methods section. LPS injection elongates escape distance (**b**) and time (**c**) without affecting average swimming speed (**d**). LPS decreases the latency to enter the dark compartment. The memory deficit induced by LPS was attenuated by BV treatment (**e**). Values are presented as mean ± S.E. from eight mice. ^#^ *p* < 0.05 compared to control, ^*^ *p* < 0.05 compared to LPS

Seven- to eight-week male imprinting control region (ICR) mice (Daehan Biolink, Chungcheongbuk-do, Korea) were maintained and handled in accordance with the humane animal care and use guidelines of the Korean FDA. All experiments were approved and carried out according to the Guidelines for the Care and Use of Animals [Animal Care Committee of Chungbuk National University, Korea (CBNUA-436-12-02)]. All efforts were made to minimize animal suffering, to reduce the number of animals used. All mice were housed in a room with automatic control of temperature (21~25 °C), relative humidity (45–65 %), and light-dark (12–12 h) cycles. To induce neuroinflammatory cognitive impairment model, LPS (2.5 mg/kg) was administrated intraperitoneally [26]. The LPS (serotype O55:B5, Sigma, St. Louis, MO. USA, final concentration of 1 mg/mL) was dissolved, and aliquots in distilled water (DW) were stored at −20 °C until use. The BV (final concentration of 1 mg/mL) was dissolved in DW, and aliquots were stored at −20 °C until use. These are the following four groups: (I) saline + saline group (control); (II) saline + LPS group (LPS); (III) BV (0.8 μg/kg) + LPS group (BV 0.8); and (IV) BV (1.6 μg/kg) + LPS group (BV 1.6), and each group was assigned to eight mice. First, intraperitoneal (i.p.) injection (0.8 μg/kg, 1.6 μg/kg) of BV and i.p. injection (2.5 mg/kg) of LPS or control (saline) was administered after 30 min. The doses of BV were the same with the previous study [23]. This step was performed daily for seven days. And then, the behavioral tests of learning and memory capacity were assessed using two separate tests (water maze and passive avoidance test). After training once beforehand (day 0), the test was performed in the date of the first, third, and seventh day (Fig. 1a).

### Morris water maze test

The water maze test is also a commonly accepted method for memory test, and we performed this test as described by Morris et al. [27]. Maze testing was fulfilled by the SMART-CS (Panlab, Barcelona, Spain) program and equipment. A circular plastic pool (height; 35 cm, diameter; 100 cm) was filled with squid-ink water kept at 22–25 °C. An escape platform (height; 14.5 cm, diameter; 4.5 cm) was submerged 1–1.5 cm below the surface of the water in position. On training trials, the mice were placed in a pool of water and allowed to remain on the platform for 10 s and were then returned to the cage. The mice that did not find the platform within 120 s were placed on the platform for 3 s at the end of the trial. Then mice stay on the platform for seven more seconds. These trials were performed on a single platform and in three starting positions of rotational starting. Escape latency, escape distance, swimming speed, and swimming pattern of each mouse was monitored by a camera above the center of the pool connected to a SMART-LD program (Panlab, Barcelona, Spain).

### Passive avoidance performance test

The passive avoidance test is generally accepted as a simple method for memory testing. The passive avoidance response was determined using a “step-through” apparatus (Med Associates Inc, Vermont, USA) that is consisted of an illuminated and a dark compartment (each 20.3 × 15.9 × 21.3 cm) adjoining each other through a small gate with a grid floor, 3.175-mm stainless steel-rod set 8 mm apart. On the first day, the mice were placed in the illuminated compartment facing away from the dark compartment for the training trial. When the mice moved completely into the dark compartment, they received an electric shock (2 mA, 3-s duration). Then the mice were returned to their case. From training day after 1, 3, and 7 days, the mice were placed in the illuminated compartment and the latency period to enter the dark compartment defined as “retention” was measured. The time when the mice entered into the dark compartment was recorded and described as step-through latency. The retention trials were set at a limit of 10 min of cutoff time.

### Brain collection and preservation

After behavioral tests, mice were perfused with phosphate-buffered saline (PBS, pH 7.4) under inhaled diethyl ether anesthetization. The brains were immediately pulled from the skull, cut, and divided into the left brain and right brain. One hippocampus was removed and stored at −80 °C, the others were fixed in 4 % paraformaldehyde for 48 h at 4 °C and transferred to 30 % sucrose solutions.

### Astrocytes and microglial BV-2 cell culture

Astrocytes were prepared from the cerebral cortex of rat embryos (E18). After removal of the exception of the cerebral cortex, the cerebral cortex was dissociated into a single-cell suspension by trypsinization and mechanical disruption. The cells were seeded on T-75 culture flasks and incubated in Dulbecco’s modified eagle medium (DMEM/F-12) (Invitrogen, Carlsbad, CA) containing 10 % fetal bovine serum (FBS) (Invitrogen). The culture medium was replaced at every 3 days thereafter. After 10–12 days, the cultures became confluent, and loosely attached microglia and oligodendrocyte precursor cells were removed from the cell monolayer through use of a shaking incubator (37 °C, 350 RPM, 2–4 h). Astrocytes were subsequently detached using trypsin-EDTA and plated into 100-mm cell culture dishes. The percentage of astrocytes in our culture system is more than 95 %. Microglial BV-2 cells were maintained with serum-supplemented culture media of DMEM supplemented with FBS (10 %) and penicillin (100 units/ml). The microglial BV-2 cells were incubated in the culture medium in a humidified incubator at 37 °C and 5 % CO2. The cultured cells were treated simultaneously with LPS (1 μg/ml) and several concentrations (0.5, 1, 2 μg/ml) of BV dissolved in distilled water, and the cells were harvested after 48 h.

### Immunohistochemical staining

After being transferred to 30 % sucrose solutions, brains were cut into 30-μm sections by using a cryostat microtome (Leica CM 1850; Leica Microsystems, Seoul, Korea). After two washes in PBS (pH 7.4) for each 10 min, endogenous peroxidase activity was quenched by incubating the samples in 3 % hydrogen peroxide in PBS for 20 min, and two washes in PBS for each 10 min. The brain sections were blocked for 1 h in 5 % bovine serum albumin (BSA) solution and incubated overnight at 4 °C with a mouse polyclonal antibody against glial fibrillary acidic protein (GFAP) (1:300; Santa Cruz Biotechnology, Inc., Santa Cruz, CA, USA), inducible nitric oxide synthase (iNOS) (1:300; Novus Biologicals, Inc., Littleton), a rabbit polyclonal antibody against cyclooxygenase-2 (COX-2) (1:300; Cell Signaling Technology, Inc., Beverly, MA, USA), and a goat polyclonal antibody against ionize calcium-binding adapter molecule 1 (IBA-1) (1:300; Abcam, Inc., Cambridge, MA, USA). After incubation with the primary antibodies, brain sections were washed thrice in PBS for each 10 min. After washing, brain sections were incubated for 1–2 h at room temperature with the biotinylated goat anti-rabbit or goat anti-mouse or donkey anti-goat IgG-horseradish peroxidase (HRP) secondary antibodies (1:500; Santa Cruz Biotechnology, Inc., Santa Cruz, CA, USA). Brain sections were washed thrice in PBS for each 10 min and visualized by chromogen DAB (Vector Laboratories) reaction for up to 10 min. Finally, brain sections were dehydrated in ethanol, cleared in xylene, mounted with Permount (Fisher Scientific, Hampton, NH), and evaluated on a light microscopy (Olympus, Tokyo, Japan) (×50 or ×200).

### Fluorescence microscopy

The fixed cells and brain sections were exposed to the following primary antibodies: GFAP (1:100, Santa Cruz Biotechnology Inc. Santa Cruz, CA, USA), IBA-1 (Abcam, Inc., Cambridge, MA, USA), and Aβ_1-42_ (1:100, Cell Signaling Technology, Inc. Beverly, MA) at room temperature for 2 h. After incubation, the cells were washed twice with ice-cold PBS and incubated with an anti-rabbit or mouse or goat secondary antibody conjugated to Alexa Fluor 488 or 568 (Invitrogen-Molecular Probes, Carlsbad, CA) at room temperature for 1 h. Immunofluorescence images were acquired using an inverted fluorescent microscope Zeiss Axiovert 200 M (Carl Zeiss, Thornwood, NY) (×200).

### Thioflavin S staining

After being transferred to 30 % sucrose solutions, brains were cut into 30-μm sections by using a cryostat microtome (Leica CM 1850; Leica Microsystems, Seoul, Korea). After washes in distilled water for 5 min, the brain sections were transferred to gelatin-coated slices and placed in 1 % thioflavin S for 5 min. After this, the brain sections were washed in distilled water then dehydrated through ascending grades of ethanol, 50, 70, 90, and 100 % ethanol for 2 min in each grade. The sections were then mounted in a mounting medium (Fluoromount™ Aqueous Mounting Medium, Sigma, St Louis, MO, USA). The thioflavin S staining was examined using a fluorescence microscope (×100).

### DAPI/TUNEL assay

The terminal deoxynucleotidyl transferase (TdT)-mediated dUTP-biotin nick end-labeling (TUNEL) assays were performed by using the in situ cell death detection kit (Roche Diagnostics GmbH, Mannheim, Germany) according to the manufacturer’s instructions. TUNEL mixture was added onto tissue sections and incubated in a humidified chamber for 60 min at 37 °C. After each step, the tissue sections were rinsed twice with PBS (pH 7.4). For DAPI staining, tissue sections were incubated for 30 min at room temperature in the dark. The cells were then observed through a fluorescence microscope Zeiss Axiovert 200 M (Carl Zeiss, Thornwood, NY). The total number of cells in the given area was determined by using DAPI nuclear staining. The apoptotic bodies (TUNEL-stained cells) were identified under a fluorescence microscope (×200) containing green-colored nuclei. The quantity of apoptotic bodies was expressed as the average number of apoptotic cells per high-power field (visible apoptotic cells/HPE).

### Measurement of Aβ_1–42_

Lysates of brain tissue were obtained through protein extraction buffer containing protease inhibitor. Aβ_1–42_ levels were determined using each specific ELISA Kit (Immuno-Biological Laboratories Co., Ltd., Takasaki-Shi, Gunma, Japan). Protein was extracted from brain tissues (hippocampus regions), cultured astrocytes, and microglial BV-2 cells using protein extraction buffer (PRO-PREPTM, Intron Biotechnology, Korea), incubated on ice for 1 h and centrifuged at 13,000*g* for 15 min at 4 °C. In brief, 100 μl of sample was added into a precoated plate and incubated overnight at 4 °C. After washing each well of the precoated plate with a washing buffer, 100 μl of labeled antibody solution was added, and the mixture was incubated for 1 h at 4 °C in the dark. After washing, chromogen was added, and the mixture was incubated for 30 min at room temperature in the dark. Finally, the resulting color was assayed at 450 nm using a microplate absorbance reader (Sunrise™, Tecan, Switzerland) after adding stop solution.

### Assay of β- and γ-secretase activities

β- and γ-secretase activity in mice brains was determined using a commercially available β- and γ-secretase activity kit (Abcam, Inc, Cambridge, MA, USA). Protein was extracted from brain tissues (hippocampus regions) using protein extraction buffer (PRO-PREP™, Intron Biotechnology, Korea), incubated on ice for 1 h, and centrifuged at 13,000*g* for 15 min at 4 °C. The supernatant was collected. A total of 50 μl of sample (total protein 100 μg) was added to each well followed by 50 μl of ×2 reaction buffer and 2 μl of β-secretase substrate incubated in the dark at 37 °C for 2 h. Fluorescence was read at excitation and emission wavelengths of 355 and 510 nm, respectively, using a Fluostar Galaxy fluorimeter (BMG Lab Technologies, Offenburg, Germany) with Felix software (BMG Lab Technologies, Offenburg, Germany). β- and γ-secretase activity is proportional to the fluorimetric reaction and is expressed as nanomole per milligram of protein per minute.

### Nuclear extraction and gel mobility shift assay

Gel mobility shift assay was conducted using a slight modification of a previously described method [28]. In brief, 10 μg of nuclear protein of astrocytes was incubated in 25 μL of the total volume of incubation buffer (10 mmol/L Tris, pH 7.5, 100 mmol/L NaCl, 1 mmol/L dithiothreitol, 4 % glycerol, 80 mg/L salmon sperm DNA) at 4 °C for 15 min followed by another 20-min incubation with 9.25 mBq [γ-^32^P] ATP-labeled oligonucleotide containing the NF-κB-binding site at room temperature. The DNA protein-binding complex was electrophoretically resolved on a 6 % nondenatured polyacrylamide gel at 150 volts for 90 min. The gels were dried and autoradiographed using Kodak MR film at −80 °C overnight.

### Pull-down assay

Melittin was conjugated with Epoxy-activated sepharose 6B (Sigma, St Louis, Missouri, United States). BV (1 mg) was dissolved in 1 mL of coupling buffer (0.1 M NaHCO_3_ and 0.5 M NaCl, pH 11). The Epoxy-activated sepharose 6B was swelled and washed in 1 mM of HCl on a sintered glass filter, then washed with a coupling buffer. Epoxy-activated sepharose 6B beads were added to the melittin containing coupling buffer and incubated at 4 °C for overnight. After washing, unoccupied binding sites were blocked with 1 M ethanolamine at 4 °C overnight. The melittin-conjugated sepharose 6B was washed with three cycles of alternating pH wash buffers (buffer 1; 0.1 M acetate and 0.5 M NaCl, pH 4; buffer 2; 0.1 M Tris-HCl and 0.5 M NaCl, pH 8). Melittin-conjugated beads were then equilibrated with a binding buffer (0.05 M Tris-HCl and 0.15 M NaCl, pH 7.5). The control unconjugated Epoxy-activated sepharose 6B beads were prepared as described above with the absence of melittin. BV-2 cell lysates were prepared in a lysis buffer of PRO-PREBP. The cell lysates (1 mg of protein) were mixed with 20 μl of melittin-conjugated sepharose 6B or sepharose 6B at 4 °C overnight. The beads were then washed three times with TBST. The bound proteins were eluted with SDS-loading buffer. The proteins were then resolved by SDS-PAGE followed by immunoblotting with antibodies against p50 (1:1000, Santa Cruz Biotechnology Inc. Santa Cruz, CA, USA).

### Molecular modeling

Docking studies between melittin and p50 of NF-κB subunit were performed using Autodock VINA [29]. Three-dimensional structures of the NF-κB-DNA complexes were retrieved from the Protein Data Bank (PDB codes; 1VKX), and melittin was retrieved from the PDB (PDB codes; 2MLT). Starting from the co-crystallized complexes, the NF-κB p50 monomer chain (p50 from 1VKX), melittin (melittin from 2MLT) for docking, were prepared using Maestro graphical interface. The grid box was centered on the p50 monomer, and the size of the grid box was adjusted to include the whole monomer. Docking experiments were performed at various exhaustiveness values of the default, 16, 24, 32, 40, and 60. Molecular graphics for the best binding model were generated using Discovery Studio Visualizer 2.0.

### Western blotting

The hippocampus tissues, treated astrocytes, and microglial BV-2 cells were homogenized with lysis buffer (PRO-PREP; iNtRON, Seongnam, Korea), incubated on ice for 30 min, and centrifuged at 13,000*g* for 15 min at 4 °C. Cytosol protein was extracted using buffer A (50 mM HEPES, pH 7.4, 10 mM KCl, 1 mM EDTA, 1 mM EGTA, 1 mM dithiothreitol, 0.1 mg/ml PMSF, 1 μg/ml pepstatin A, 1 mg/ml leupeptin, 10 μg/ml soybean trypsin inhibitor, 10 μg/ml aprotinin, and 0.5 % Nonidet P-40), incubated on ice for 10–15 min, and centrifuged at 6000*g* for 10 min at 4 °C. This supernatant is cytosol extract. Buffer C (buffer A + 10 % glycerol and 400 mM KCl) was added to the pellet, incubated on ice for 30 min, and centrifuged at 13,000*g* for 15 min at 4 °C. This supernatant is nuclear extract. An equal amount of total protein (40 μg) was resolved on 6–15 % sodium dodecyl sulfate polyacrylamide gel and then transferred to a nitrocellulose membrane (Hybond ECL; Amersham Pharmacia Biotech, Piscataway, NJ, USA). The membranes were blocked for 1 h in 5 % skim milk solution and incubated overnight at 4 °C with specific antibodies. To detect target proteins, specific antibodies against APP, iNOS (1:1000, Novus Biologicals, Inc., Littleton), BACE1, IBA-1 (1:1000, Abcam, Inc., Cambridge, MA, USA), COX-2 (1:1000, Cell Signaling Technology, Inc., Beverly, MA, USA), GFAP, p50, p65, IκB, phospho-IκB, β-actin, and Histone H1 (1:1000, Santa Cruz Biotechnology Inc. Santa Cruz, CA, USA) were used. The blots were then incubated with the corresponding conjugated goat anti-rabbit or goat anti-mouse or donkey anti-goat IgG-horseradish peroxidase (HRP) (1:5000; Santa Cruz Biotechnology Inc. Santa Cruz, CA, USA) secondary antibodies. Immunoreactive proteins were detected with an enhanced chemiluminescence western blotting detection system. The relative density of the protein bands was scanned by densitometry using MyImage (SLB, Seoul, Korea) and quantified by Labworks 4.0 software (UVP Inc., Upland, CA, USA).

### Statistical analysis

Measurement of the image data used ImageJ (Wayne Rasband, National Institutes of Health, Bethesda, MD). Statistical analysis of the data was carried out using analysis of variance (ANOVA) for repeated measures followed by Dunnett’s post-hoc analysis using GraphPad Prism 4 software (Version 4.03, GraphPad software, Inc., La Jolla, USA).

## Results

### Inhibitory effect of BV on LPS-induced memory defects

Seven- to eight-week old ICR mice were administered with LPS (2.5 mg/kg) for 7 days to study whether BV could improve memory in LPS-induced AD mice. We used the Morris water maze and passive avoidance performance tests. The mice were trained for three trials per day. Control mice took short escape latency to get to the platform, but LPS-treated mice arrived at the location of the platform more slowly than the control mice, suggesting that the memory deficiency could be induced by LPS as reported previously [30]. BV-treated mice however, exhibited shorter escape latency than LPS-treated mice. BV-treated mice with a higher dose showed shorter escape latency than BV-treated mice with a lower dose (Fig. 1b). BV-treated mice also showed shorter escape distance compared to LPS-treated mice (Fig. 1c). However, there was no significant difference in average velocity between LPS-treated mice and BV-treated mice (Fig. 1d). Next, we examined the passive avoidance performance test 1 day after the Morris water maze test. Stay time in the white chamber indicated maintenance of memory capacity. LPS-treated mice (351.1 ± 72.9 s) stayed for a shorter time than control mice (502.0 ± 52.7 s). BV-treated mice (BV 0.8 μg/kg; 495.6 ± 46.2 s, BV 1.6 μg/kg; 454.6 ± 73.3 s) stayed for a longer time than LPS-treated mice but shorter than control mice demonstrating memory recovery effect (Fig. 1e).

### Inhibitory effect of BV in LPS-induced amyloidogenesis and Aβ accumulation

**Fig. 2:**
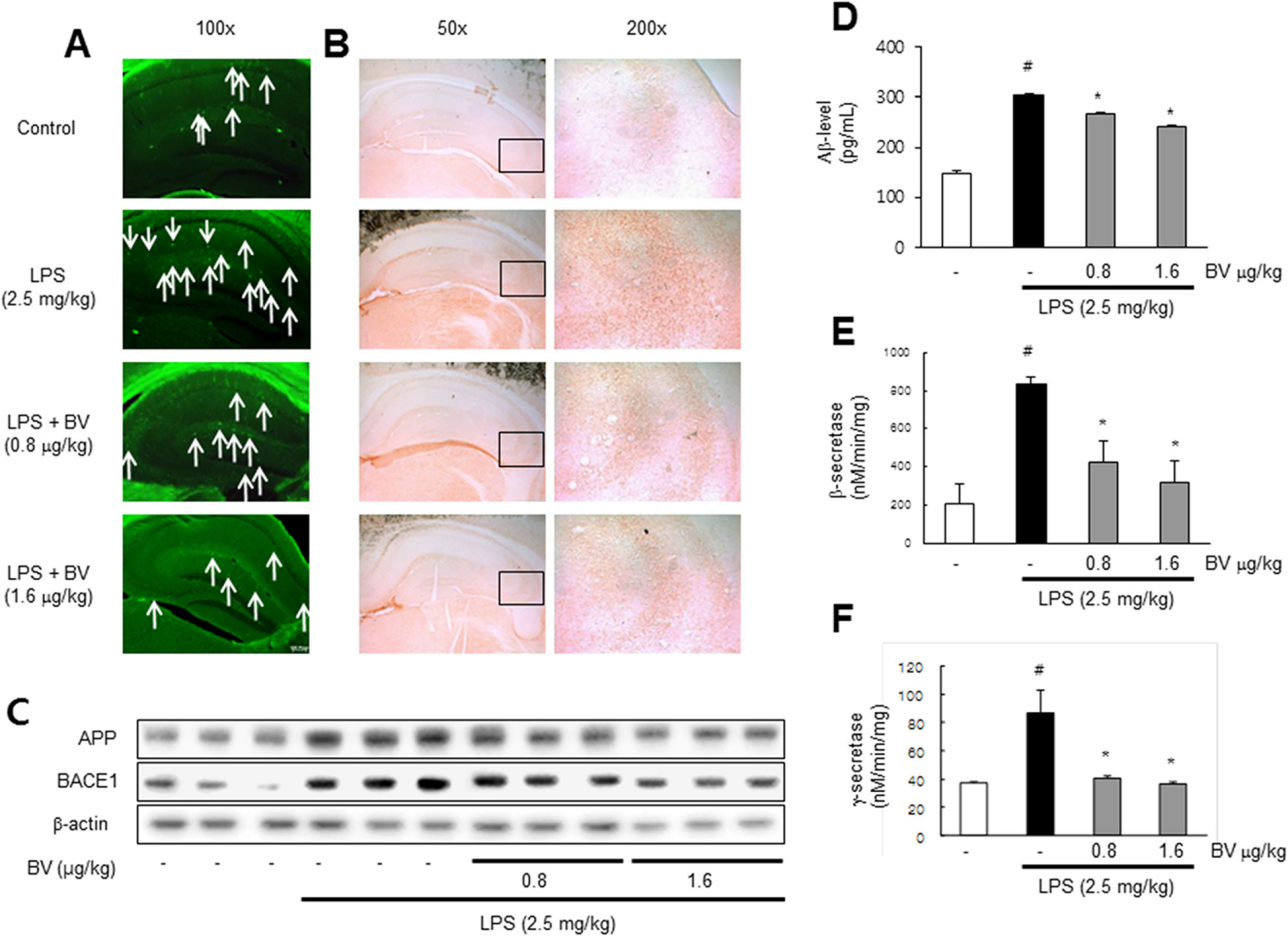
Inhibitory effect of BV on LPS-induced Aβ accumulation and amyloidogenesis in mice brain. Thioflavin S staining for detection of Aβ accumulation that represents the *arrow* in the graph (**a**). Immunostaining of Aβ protein in the hippocampus was performed in 30-μm-thick sections of mice brain with anti-Aβ_1-42_ primary antibody and the biotinylated secondary antibody (**b**). The expression of APP and BACE1 were detected by western blotting using specific antibodies in the mice brain. Each blot is representative of three experiments (**c**). The levels of Aβ_1-42_ in mice brain were measured by ELISA (**d**). Inhibitory effect of BV on LPS-induced alteration in secretase activity. The activity of β- and γ-secretase in mice brain were investigated using assay kit (**e**, **f**). Values are presented as mean ± S.E. from eight mice. ^#^ *p* < 0.05 compare to control, *p < 0.05 compared to LPS

**Fig. 3:**
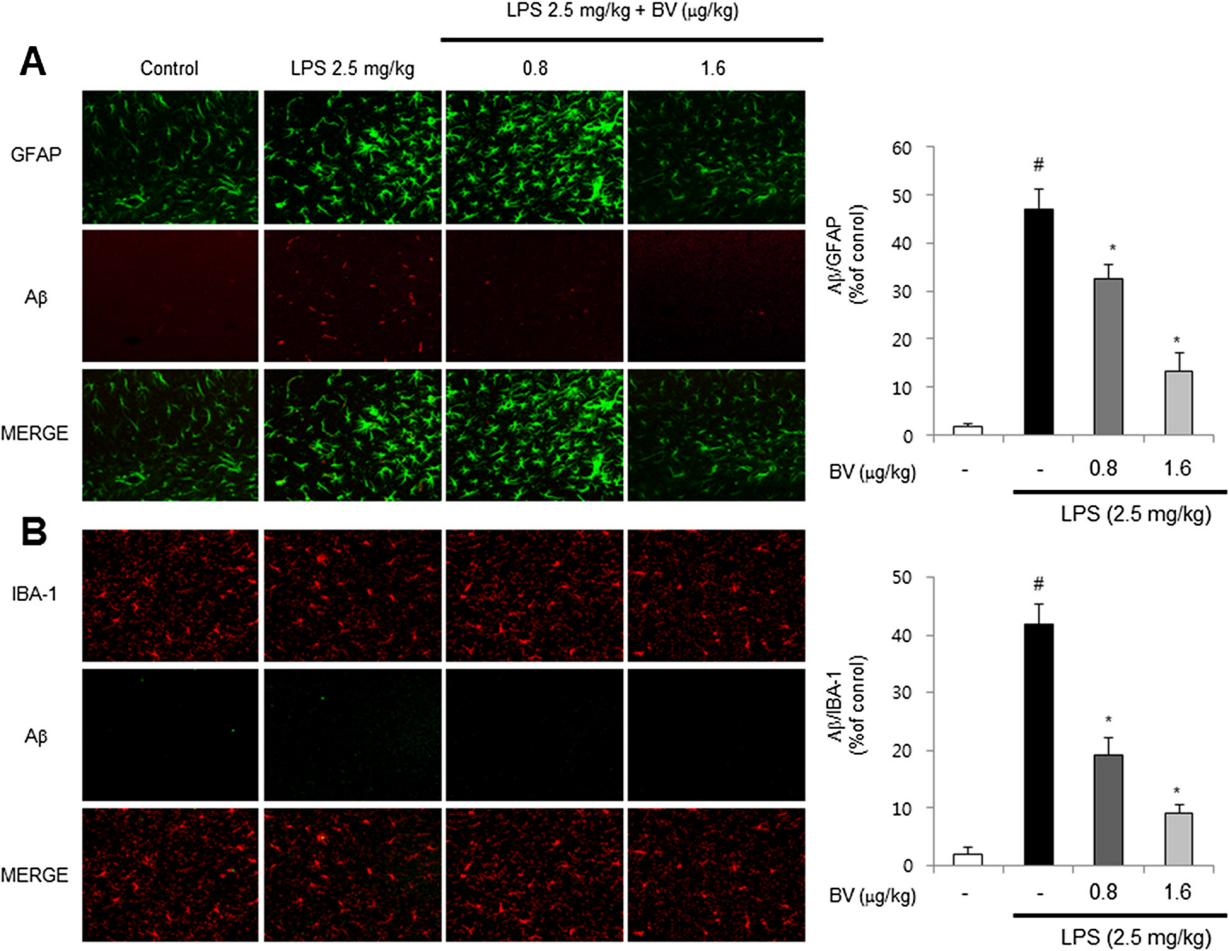
Inhibitory effect of BV on LPS-induced expression of Aβ_1-42_ in both GFAP and IBA-1-positive mice brain. Staining was performed in 30-μm-thick sections of mice brain. Confocal microscope observation was performed as described in the Methods section. Immunostaining of GFAP (*green*) and Aβ_1−42_ (*red*) protein in the hippocampus was performed with specific primary antibodies, and fluorescence was developed using Alexa 488-conjugated anti-goat and Alexa 568-conjugated anti-rabbit secondary antibodies (**a**). IBA-1 (*red*) and Aβ_1−42_ (*green*) protein in the hippocampus was performed with specific primary antibodies, and fluorescence was developed using Alexa 488-conjugated anti-mouse and Alexa 568-conjugated anti-rabbit secondary antibodies (**b**). Values are presented as mean ± S.E. from eight mice. ^#^ *p* < 0.05 compared to control, *p < 0.05 compared to LPS

AD is known to be correlated with accumulation of Aβ peptides. Therefore, we examined whether BV reduced LPS-induced Aβ accumulation and amyloidogenesis, and thus recovery memory function. Thioflavin S staining is dyed with beta sheet-rich structures of Aβ. This thioflavin S staining was used for detection of Aβ accumulation. The higher accumulation of Aβ was seen in the brain of LPS-treated mice; however, lower accumulation of Aβ was seen in the BV-treated mice brain (Fig. 2a). We also used immunohistochemistry for detection of Aβ expression and acquired the same results as thioflavin S staining (Fig. 2b). We analyzed the expression of APP and BACE1 by western blotting. LPS-treated mice increased the expression of these proteins, but BV-treated mice showed decreased expressions (Fig. 2c). To clarify how amyloidogenesis was reduced by BV, we analyzed the amount of Aβ_1−42_ level and β- and γ-secretase activities in whole brain. The level of Aβ_1−42_ and activities of β- and γ-secretase were significantly increased in LPS-treated mice brain, while these level and activities were decreased in BV-treated mice brain (Fig. 2d–f). Because the activation of astrocytes is implicated in the activation of β-secretase, we investigated whether the numbers of astrocytes (GFAP-positive cells) and the accumulation of Aβ are concomitantly increased by LPS, and whether BV reduces astrocytes activation, thereby reducing Aβ_1-42_ levels. The immunoreactive cells for both GFAP and Aβ_1-42_ were identified using a double immunofluorescence method. The co-reactive cell number for both markers was increased by LPS, but was lowered by BV treatment (Fig. 3a). In addition, we further studied into microglia cells. The double positive cell number for Aβ accumulation in microglia cells (IBA-1-positive cells) was increased by LPS, which was also lowered by BV treatment (Fig. 3b).

### Inhibitory effect of BV on LPS-induced neuronal cell death and neuroinflammation

**Fig. 4:**
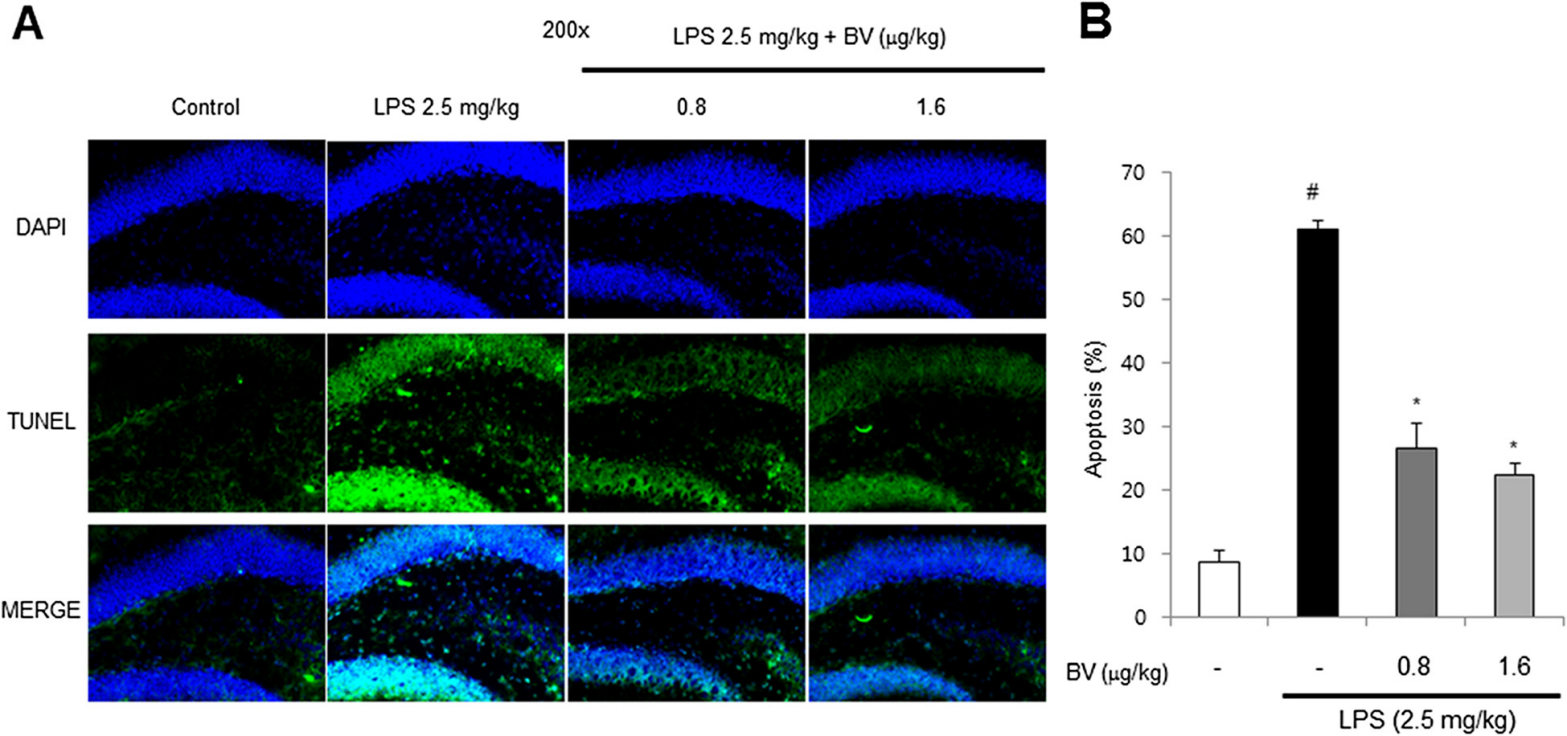
Inhibitory effect of BV on LPS-induced cell death. DAPI/TUNEL assay for detection of apoptotic cell death in the hippocampal DG zone was performed in 30-μm-thick sections of mice brain (**a**). Apoptosis (%) was defined as the percentage of the number of TUNEL-positive cells per surface of unit (**b**). Values are mean ± S.E. (*n* = 8). ^#^ *p* < 0.05 compared to control, *p < 0.05 compared to LPS

To observe death of brain cells, we used the DAPI/TUNEL assay. Apoptosis (%) was defined as the percentage of the number of TUNEL-positive cells per surface of unit. TUNEL-stained brain cells were more in LPS-treated mice brain (61.0±1.8) compared to control (8.6±1.4) and BV-treated mice brain. BV-treated mice brain values were 26.6±4.1 (0.8 μg/kg), and 22.3±1.7 (1.6 μg/kg), respectively (Fig. 4).

**Fig. 5:**
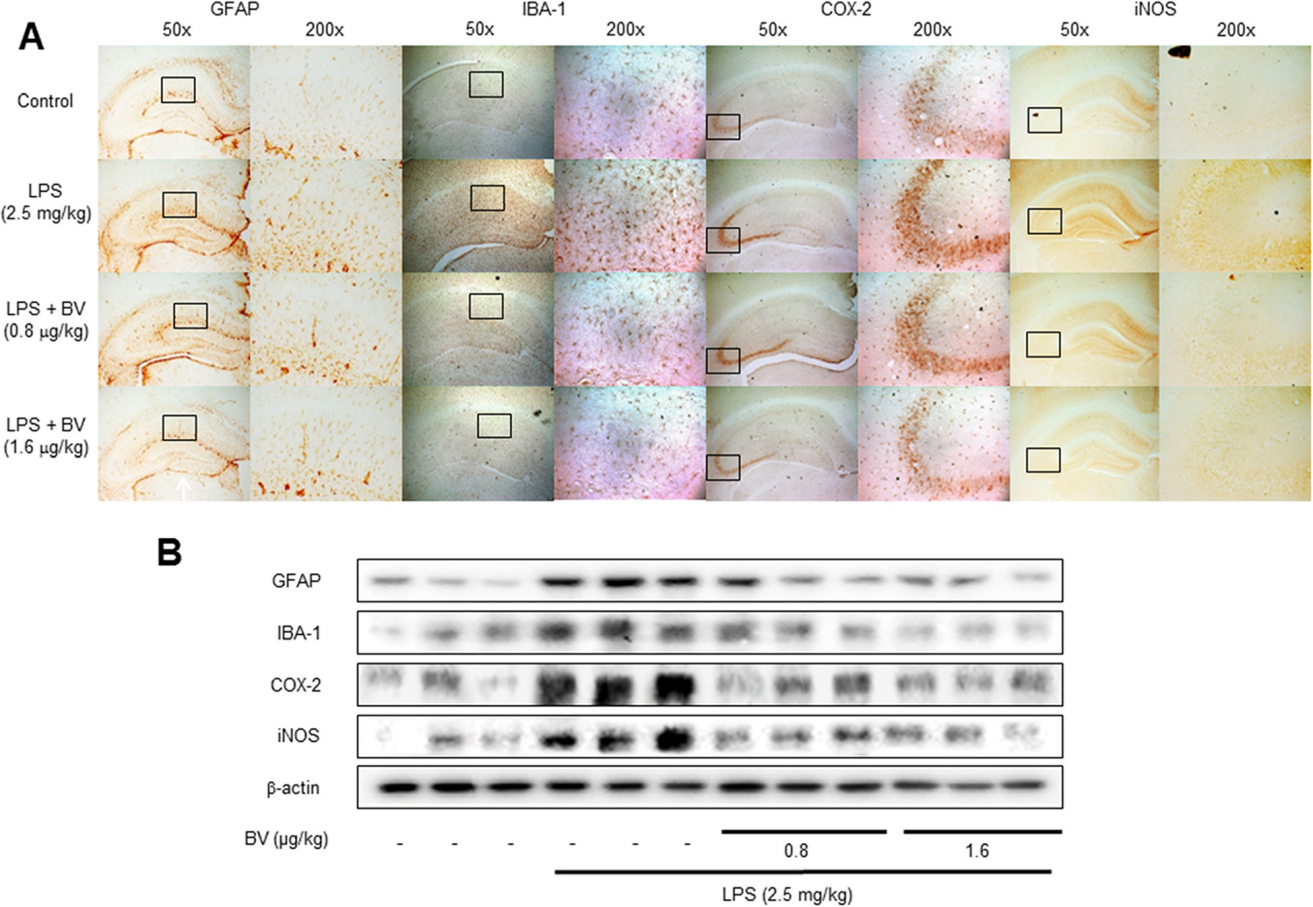
Inhibitory effect of BV on LPS-induced brain cell activation and neuroinflammation. Immunostaining of GFAP, IBA-1, COX-2, and iNOS proteins in the hippocampus were performed in 30-μm-thick sections of mice brain with specific primary antibodies and the biotinylated secondary antibodies (*n* = 8) (**a**). The expression of GFAP, IBA-1, COX-2, and iNOS were detected by western blotting using specific antibodies in the mice brain. Each blot is representative of three experiments (**b**)

Activated NF-κB by LPS can trigger neuroinflammation in neuronal cells through activation of inflammatory cells such as astrocytes and microglia cells. To confirm activation of astrocytes and microglia cells, we used the immunohistochemistry and western blotting to detect the expression of GFAP (a marker of astrocyte activation), IBA-1 (a marker of microglia cell activation), iNOS, and COX-2 in the brain. The results showed that LPS-treated mice brain increased expression of these proteins in comparison with control; however, expression of these proteins was decreased in BV-treated mice brain (Fig. 5a, b).

### Inhibitory effect of BV on LPS-induced NF-κB activation

**Fig. 6:**
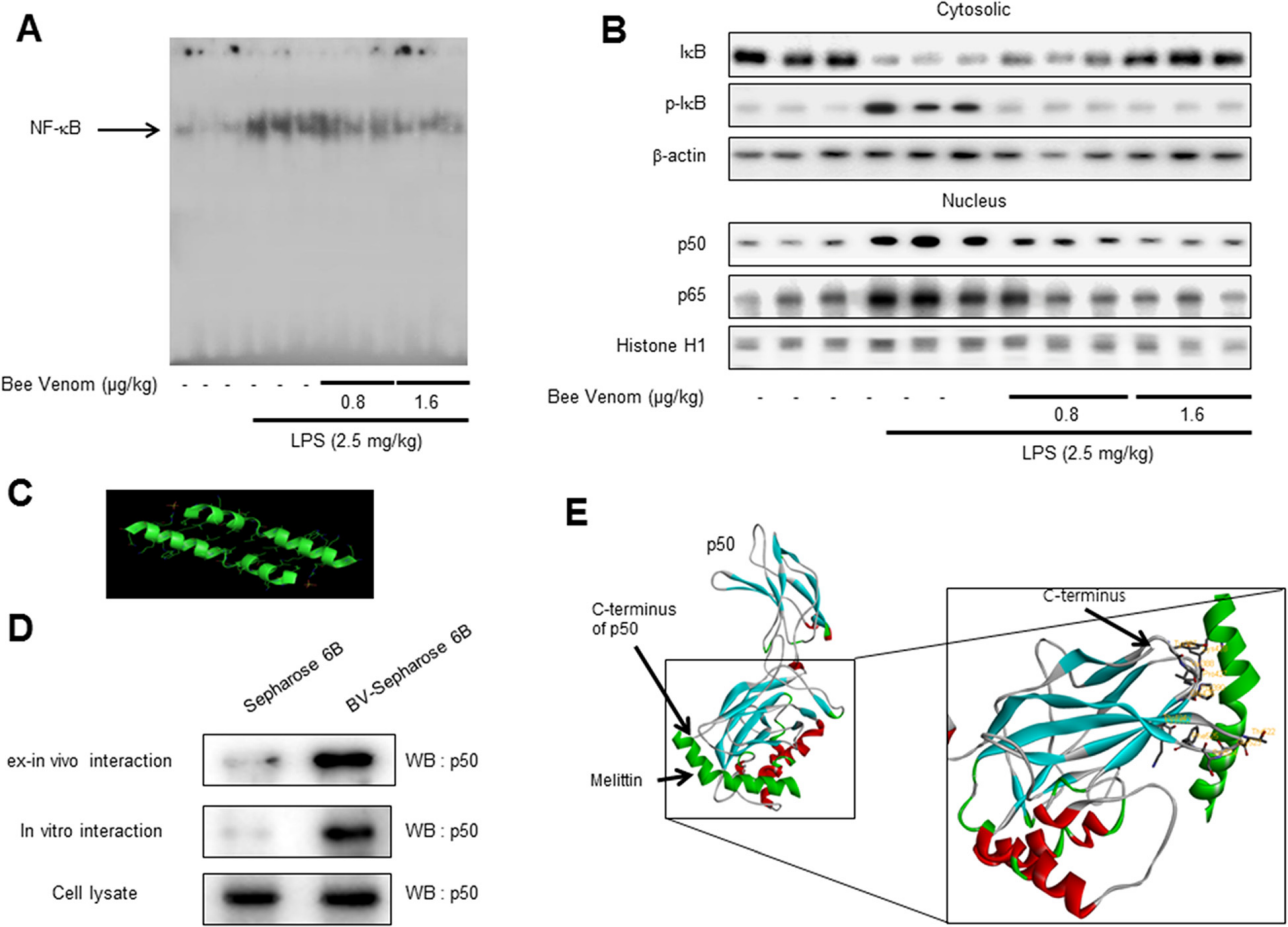
Inhibitory effect of BV on LPS-induced NF-κB DNA-binding activity and on NF-κB-related protein in mice brain. Activation of NF-κB was investigated using EMSA as described in the Methods section. Nuclear extracts were subjected to DNA-binding reaction with ^32^P end-labeled oligonucleotide specific to NF-κB. Specific DNA binding of the NF-κB complex is indicated by an *arrow* (**a**). The expression of IκB, p-IκB, p50, and p65 were detected by western blotting using specific antibodies in the mice brain. Each blot is representative of three experiments (**b**). Structural interaction between melittin and p50 of NF-κB subunit. Melittin structure is **c**. Pull-down assay identifies an interaction between the melittin and p50. Melittin was conjugated with melittin-activated Sepharose 6B (**d**). The docking model of melittin with p50 is as described in the Materials and Methods section (**e**)

To determine whether BV is able to inhibit LPS-induced DNA-binding activity of NF-κB in the mice model, nuclear extracts from mice brain were prepared and assayed for NF-κB DNA-binding activity by EMSA (Fig. 6a). LPS significantly induced NF-κB; however, its activity was effectively blocked by BV. When treated with LPS, we found that NF-κB proteins (p50 and p65) were translocated to the nucleus, and IκB was phosphorylated, while BV remarkably inhibited LPS-induced translocation of p50 and p65 in the nucleus as well as IκB phosphorylation in a dose-dependent manner in mice brain (Fig. 6b).

### Interaction between melittin and p50 of the NF-κB subunit

To investigate whether the inhibitory effect of BV on activity due to direct binding of melittin, a major component of BV (Fig. 6c), and p50, a subunit of NF-κB, we performed a pull-down assay and molecular docking experiment between melittin and p50 of the NF-κB subunit using melittin-sepharose 4B beads. The binding of melittin to p50 was then detected by immunoblotting with an anti-p50 antibody. The results indicated that melittin interacted with cell lysates containing p50 from microglial BV-2 cells as well as recombinant p50 protein (Fig. 6d). To identity the binding site of p50 to melittin, we performed computational docking experiments with melittin and p50. The binding study was performed using Autodock Vina software and showed that melittin binded near to the C-terminus of p50 (melittin binds to Tyr387, Vol388, Pro39, Lys392, Thr424, Pro427, Lys428, Thr522, Ser524, and Phe525 of p50) where nuclear localization signal (NLS) is located in this specific p50 (Fig. 6e).

### Inhibitory effect of BV on amyloidogenesis and neuroinflammatory responses in astrocytes and microglia cells

**Fig. 7:**
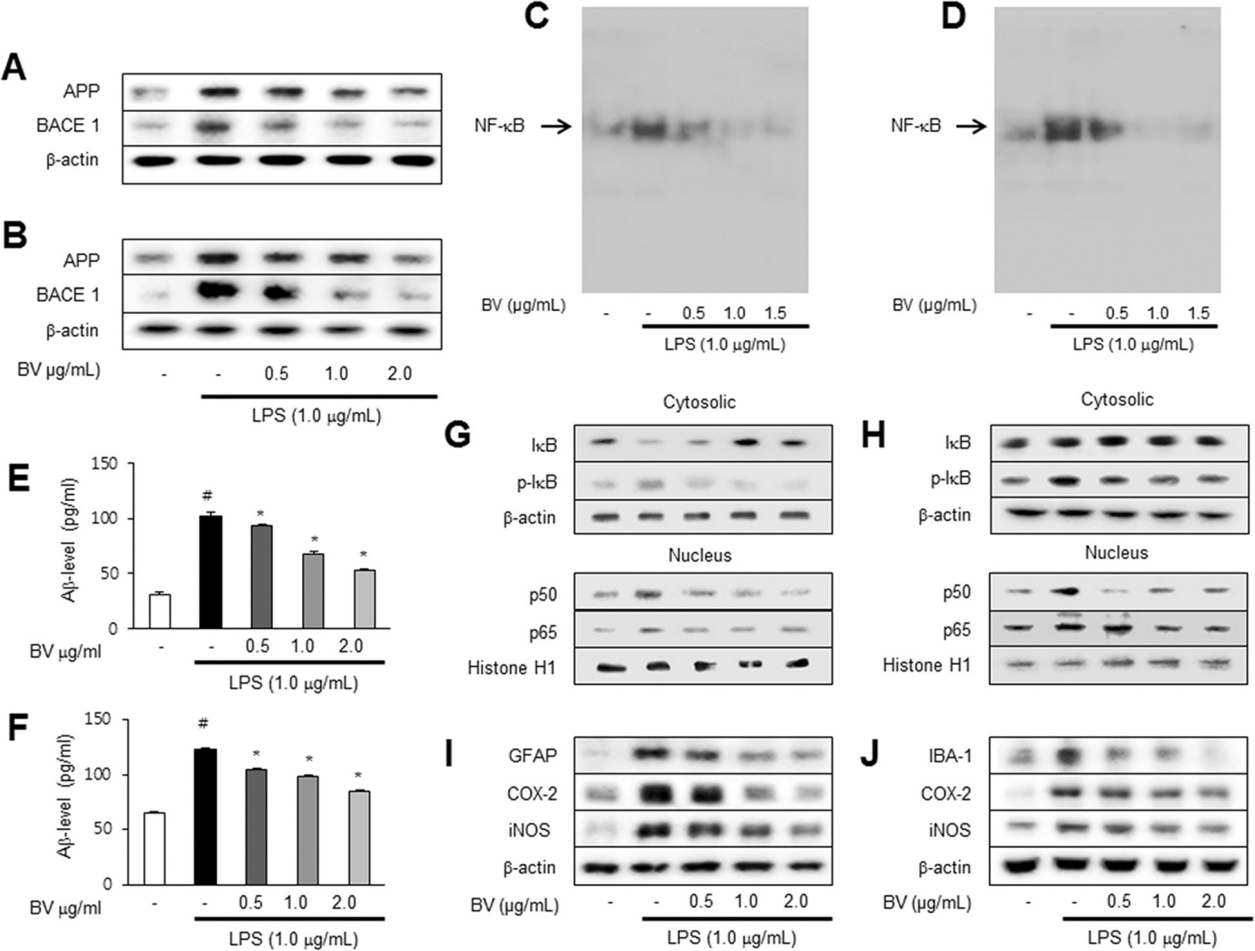
The expression of APP and BACE1 in astrocyte primary cells (**a**) and microglia BV-2 cells (**b**) were detected by western blotting using specific antibodies. Inhibitory effect of BV on LPS-induced NF-κB DNA-binding activity and on NF-κB-related protein in astrocyte primary cells (**c**) and microglia BV-2 cells (**d**). Activation of NF-κB was investigated using EMSA as described in the Methods section. Nuclear extracts were subjected to DNA-binding reaction with ^32^P end-labeled oligonucleotide specific to NF-κB. Specific DNA binding of the NF-κB complex is indicated by an *arrow*. The levels of Aβ_1-42_ in astrocyte primary cells (**e**) and microglia BV-2 cells (**f**) were measured by ELISA (*n* = 3). Values are presented as mean ± S.E. of the three independent experiments performed in triplicate. ^#^ *p* < 0.05 compared to control, *p < 0.05 compared to LPS. The expression of IκB, p-IκB, p50, and p65 in astrocyte primary cells (**g**) and microglia BV-2 cells (**h**) was detected by western blotting using specific antibodies. The expression of GFAP, IBA-1, COX-2, and iNOS in astrocyte primary cells (**i**) and microglia BV-2 cells (**j**) were detected by western blotting using specific antibodies. Each blot is representative of three experiments

**Fig. 8:**
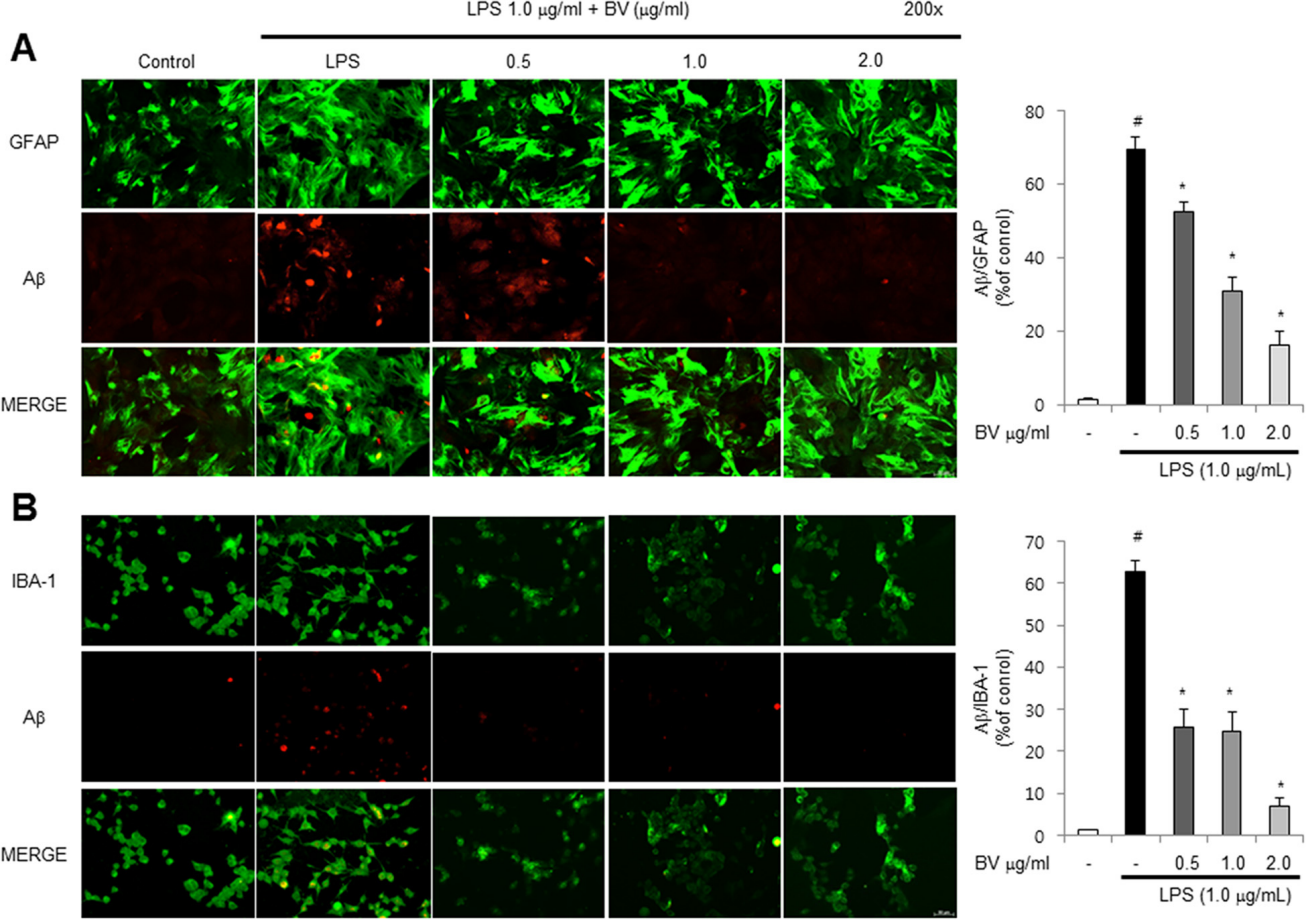
The cultured astrocytes were incubated with anti-GFAP (*green*) and anti- Aβ_1−42_ (*red*) primary antibodies, and microglial BV-2 cells were incubated with anti-IBA-1 (*green*) and anti- Aβ_1−42_ (*red*) primary antibodies. Fluorescence was developed using Alexa 488-conjugated anti-mouse and goat and Alexa 568-conjugated anti-rabbit secondary antibodies (**a**) and (**b**). Values are presented as mean ± S.E. from three mice. ^#^ *p* < 0.05 compared to control, *p < 0.05 compared to LPS

To find anti-amyloidogenesis of BV in primary cultured astrocytes (Fig. 7a) and microglial BV-2 cells, both cells were treated with 1 μg/mL of LPS and 0.5, 1 and 2 μg/mL of BV (Fig. 7b) and then detected APP and BACE1 expression by western blotting. LPS-treated cells increased expression of APP and BACE1 than control, but BV-treated cells clearly decreased expression of APP and BACE1 than LPS-treated cells. Moreover, the LPS-induced Aβ_1-42_ level was decreased by the treatment of BV determined by ELISA (Fig. 7c, d). We also discovered that BV inhibited LPS-induced NF-κB activity, translocation of NF-κB proteins (p50 and p65) to the nucleus and IκB phosphorylation in both cells (Fig. 7g, h). The expression of inflammatory proteins (iNOS, COX-2, GFAP, and IBA-1) was also detected by western blotting using specific antibodies. The BV attenuated LPS-induced increased expression of inflammatory proteins in a dose-dependent manner (Fig. 7i, j). On the same principle as brain sections, we detected double immunofluorescence in both cells by confocal microscope analysis. Co-expression of GFAP (astrocytes marker) and Aβ_1-42_ was increased by LPS but was decreased by BV treatment in primary cultured astrocytes (Fig. 8a). Co-expression of IBA-1 (microglia cell marker) and Aβ_1-42_ in microglial BV-2 cells showed the same results as primary cultured astrocytes (Fig. 8b).

## Discussion

Increasing Aβ accumulation and neuroinflammation in the brain contributes to the onset development of AD by causing neuronal cell death [2, 3]. Our study showed that BV prevented LPS-induced memory impairment. Significantly, BV ameliorated LPS-mediated amyloidogenesis and neuroinflammatory reactions in the brain as well as cultured inflammatory neuronal cells, thus memory improvement through direct inhibition of NF-κB. These data indicate that BV could be useful for treatment of AD. Our previous study revealed that LPS injection caused amyloidogenesis and memory loss in mice brain through NF-κB activation [30]. The aging process is a potential contributor to the accumulation of Aβ and other risk factors of AD. Recent study also demonstrated that the neuroinflammatory response to surgery causes postoperative cognitive dysfunction via the activation of AD pathogenic mechanisms such as gliosis, microgliosis, astrogliosis, enhanced production of Aβ, and τ protein phosphorylation in old subjects [31]. For these reasons, the elder mice are more effective for the study of AD pathology. But, in the present study, we used seven- to eight-week old mice because we already showed that the AD pathogical changes were found in 7–10 weeks after treatment of LPS (2.5 mg/kg) [26, 30, 32, 33]. NF-κB activates the transcription of APP, BACE1, and γ-secretase and therefore increases generation of Aβ because NF-κB is a transcription factor of these genes [13, 34]. Our recent studies demonstrated that melittin, a major component of BV, inhibited LPS-induced NF-κB activation by interrupting p50 translocation through interaction with sulfhydryl residue of p50 or IKK [23, 24]. Our present study also showed that BV reduced LPS-induced DNA-binding activities of NF-κB, interrupting p50 and p65 translocation through preventing IκB phosphorylation in mice brain as well as cultured astrocytes and microglia cells. Thus, the inhibiting effect of BV on NF-κB activity could result in anti-amyloidogenesis. Similar to the present study, our previous studies also showed that thiacremonone and 4-O-methylhonokiol inhibited transcriptional and DNA-binding activity of NF-κB via inhibition of IκB degradation as well as p50 and p65 translocation into the nucleus of the brain and cultured astrocytes and microglial BV-2 cells, and thus these compounds enhance memory function by anti-amyloidogenic effects [14, 30]. Huang X. et al. showed inhibition of the accumulation of Aβ through inhibition of IKK activation, IκB degradation, and the subsequent NF-κB activation [35]. These results demonstrated that direct binding of melittin, a major component of BV, to p50 results in the inhibitory effect of BV on NF-κB, and inhibition of NF-κB could be significant for the anti-amyloidogenic effect of BV.

Anti-amyloidogenesis effect of BV was associated with decreases of expression of inflammatory response genes such as iNOS and COX-2. Guo et al. demonstrated that neuroinflammation plays an important role in the process of amyloid deposition [36]. Deng et al. also showed that LPS-induced neuroinflammation was associated with amyloidogenesis in adult rat brains [37]. Neuroinflammation is associated with a broad spectrum of neurodegenerative diseases including AD [38]. It has been shown that there are increases in markers of neuroinflammation in AD patients’ brain [39]. LPS-induced systemic inflammation has been shown to impair neuronal cells in animal models for AD [9, 30, 40]. Actually, LPS kills neurons after treatment of high concentrations and long-term periods of LPS. In fact, the induction of neuroinflammation or brain cell death depends on the experimental injection method, concentration, and periods of LPS treatment. Recent study demonstrated that long-term sustainable (16th day after LPS injections) activation of astroglial NF-kappaB following systemic inflammation by LPS (10 mg/kg) was associated with brain cell and microvasculature injury in the sub-region of the hippocampus which ultimately likely results in brain functional impairment [41]. Lee et al. demonstrated that two different doses of LPS (0.25 or 0.75 mg/kg/day for 7 days) could induce memory dysfunction and amyloidogenesis but did not show significant difference between the two doses (Y.J. Lee, J.A. Kim and J.T. Hong, unpublished observations). We also observed the low concentrations of LPS (0.25 mg/kg/day) for 3 to 7 days and found that they were really not much different between 3 and 7 days [9]. It has also been demonstrated that several compounds such as 4-O-methylhonokiol, obovatol, and thiacremonone showed anti-amyloidogenesis and memory-recovering effect in LPS (0.25 mg/kg/day for 7 days)-induced amyloidogenesis and memory loss [14, 30, 32]. Based on these previous data, we used 2.5 mg/kg for 7 days for the treatment of mice for this present study. Sheng et al. showed that LPS-induced neuroinflammation of APPswe transgenic mice increases levels of APP and Aβ and causes the accumulation of Aβ within the cell bodies of neurons. Neuroinflammation can be caused by excessive activation of astrocytes and microglia cells. Lee et al. showed that activated astrocytes and microglial BV-2 cells by LPS produced TNF-α, IL-1β, iNOS, and COX-2 in cultured astrocytes and microglial BV-2 cells [42]. It was also reported that the expression of beta APP was elevated by activation of astrocytes and microglia cells in primary cultures of a cerebral cortex from a newborn rat [43]. Thus, inhibition of LPS-induced of neuroinflammation by inactivation of astrocytes and microglia cells by BV could be associated with anti-amyloidogenesis of BV. Astrocytes and microglia cells are activated by the activation of NF-κB [44]. Rolova et al. showed that cultured microglia deficient for the gene (Nfkb1) encoding p50 subunit shows reduced induction of proinflammatory mediators, increased expression of anti-inflammatory genes in response to LPS exposure in microglia cells. They also showed that p50 NF-κB^−/−^mice (Nfkb1-deficient mice) crossed with transgenic Alzheimer mice (APdE9 transgenic mice) reduced Aβ levels [45]. It was proved that NF-κB is directly involved in the regulation of GFAP gene expression in cultured primary human brain astrocytes [46]. Webster et al. showed decrease in translocation of p65 in genetic knock-down in vitro via siRNA, or in vivo P2Y_12_, microglial purinergic receptor transgenic mice (P2Y_12_^−/−^, P2Y_12_^+/−^) [47]. Wang et al. also showed that 3-N-butylphthalide (NBP) has been reported to attenuate astroglial activation and exert neuroprotective effects in AD transgenic mice by attenuation of Aβ-induced activation of astrocytes and neuroinflammation via inhibition of the NF-κB signaling pathway [48, 49]. Our various AD animal models have shown that activity of NF-κB increased activation of both cells [14, 30, 50, 51]. These data suggest that blocking of NF-κB is also significant for inactivation of astrocytes and microglia cells for preventing neuroinflammation and amyloidogenesis.

The way to inhibit NF-κB by BV is important for the basic principle for inhibition of NF-κB. In this regard, it is noteworthy that BV binds to protein of p50, a subunit of NF-κB evidenced by pull-down assay and docking model. 7,8-dihydroxy-4-methylcoumarin (7,8-DHMC), 5,7-dihydroxy-4-methylcoumarin (5,7-DHMC) and gallic acid have shown that direct binding to NF-κB subunits could result in inhibition of NF-κB activities. 7,8-DHMC and 5,7-DHMC form three hydrogen bonds with DNA-binding region (DBR), but gallic acid makes five hydrogen bonds with DBR [52]. We previously found that the inhibitory effect of BV on NF-κB due to direct binding of melittin, a major component of BV to p50 subunit (DNA-binding site) of NF-κB, was determined by surface plasmon resonance analyzer [23]. Our present study confirmed that melittin binds to p50 of the NF-κB subunit through the docking model and pull-down assay. However, unlike previous findings, the present study showed that melittin directly binds to the C-terminus of p50 (melittin binds to Tyr387, Vol388, Pro39, Lys392, Thr424, Pro427, Lys428, Thr522, Ser524, and Phe525 of p50) where nuclear localization sequence (NLS) is located. It was reported that blocking NLS is important for inhibition of NF-κB activity by suppressing nuclear translocation of p50 [53]. Melittin also binds to the dimerization interface of p50 (melittin binds to Arg552, Aso554, Glu565, Tyr567, Leu569, His604, Arg605, Phe607, Val610, and Lys612 of p50); therefore, melittin potentially prevents p50-p65 dimerization, and melittin also can interrupt the nuclear translocation of p50. These data indicated that BV could directly bind to NF-κB (p50), thus inhibiting NF-κB activity.

BV has demonstrated the inhibitory effect on rheumatoid arthritis (RA) in a carrageenan rat model and a rat model of chronic adjuvant-induced arthritis, amyotrophic lateral sclerosis (ALS) in a hSOD1G93A mutant mice model, and Parkinson’s disease (PD) in a 1-methyl-4-phenyl-1,2,3,6-tetrahydropyridine (MPTP)-induced mice model through control of NF-κB activation [23, 54, 55]. BV also has shown the cancer cell growth inhibitory effect via inactivation of NF-κB in non-small cell lung cancer (NSCLS) cells and human prostate cancer cells (LNCaP, DU145, and PC-3) [56, 57]. Moreover, BV and melittin inhibited cell proliferation and induced apoptosis in rat aortic vascular smooth muscle cell (VSMC) via suppressions of NF-κB activation [58]. These studies showed that BV could be useful for treatment of several diseases through inhibition of NF-κB activation. To conclude, our present data indicate that BV could be effective for treatment and/or prevention of the development of diseases such as AD through anti-amyloidogenesis and anti-inflammatory responses by inhibiting NF-κB activation.

## Conclusions

We demonstrated that BV decreased amyloidogenesis and neuroinflammation. This observation was confirmed by expression of GFAP, IBA-1, COX-2, and iNOS. These proteins were decreased in BV injection groups. We also observed that BV has effect on the inhibition of NF-κB activation through expression of IκB, phospho-IκB, p50, and p65. Especially, melittin, a major component of BV, directly binds to the C-terminus of p50 where NLS is located, so they interrupt translocation of NF-κB. Therefore, BV can be used as a treatment of amyloidogenesis and neuroinflammation disease such as AD and disease by NF-κB activation.
